# Rapid Implementation of an Integrated Large-Scale HIV Counseling and Testing, Malaria, and Diarrhea Prevention Campaign in Rural Kenya

**DOI:** 10.1371/journal.pone.0012435

**Published:** 2010-08-26

**Authors:** Eric Lugada, Debra Millar, John Haskew, Mark Grabowsky, Navneet Garg, Mikkel Vestergaard, James Kahn, Nicholas Muraguri, Jonathan Mermin

**Affiliations:** 1 CHF International, Nairobi, Kenya; 2 CHF International, Silver Spring, Maryland, United States of America; 3 Liverpool School of Tropical Medicine, Liverpool, United Kingdom; 4 ESP/UN Foundation, Washington, D.C., United States of America; 5 Vestergaard-Frandsen, Inc., Lausanne, Switzerland; 6 University of California San Francisco, San Francisco, California, United States of America; 7 National AIDS/STI Control Program (NASCOP), Ministry of Public Health and Sanitation, Nairobi, Kenya; 8 Coordinating Office for Global Health, Centers for Disease Control and Prevention, Nairobi, Kenya; BMSI-A*STAR, Singapore

## Abstract

**Background:**

Integrated disease prevention in low resource settings can increase coverage, equity and efficiency in controlling high burden infectious diseases. A public-private partnership with the Ministry of Health, CDC, Vestergaard Frandsen and CHF International implemented a one-week integrated multi-disease prevention campaign.

**Method:**

Residents of Lurambi, Western Kenya were eligible for participation. The aim was to offer services to at least 80% of those aged 15–49. 31 temporary sites in strategically dispersed locations offered: HIV counseling and testing, 60 male condoms, an insecticide-treated bednet, a household water filter for women or an individual filter for men, and for those testing positive, a 3-month supply of cotrimoxazole and referral for follow-up care and treatment.

**Findings:**

Over 7 days, 47,311 people attended the campaign with a 96% uptake of the multi-disease preventive package. Of these, 99.7% were tested for HIV (87% in the target 15–49 age group); 80% had previously never tested. 4% of those tested were positive, 61% were women (5% of women and 3% of men), 6% had median CD4 counts of 541 cell/µL (IQR; 356, 754). 386 certified counselors attended to an average 17 participants per day, consistent with recommended national figures for mass campaigns. Among women, HIV infection varied by age, and was more likely with an ended marriage (e.g. widowed vs. never married, OR.3.91; 95% CI. 2.87–5.34), and lack of occupation. In men, quantitatively stronger relationships were found (e.g. widowed vs. never married, OR.7.0; 95% CI. 3.5–13.9). Always using condoms with a non-steady partner was more common among HIV-infected women participants who knew their status compared to those who did not (OR.5.4 95% CI. 2.3–12.8).

**Conclusion:**

Through integrated campaigns it is feasible to efficiently cover large proportions of eligible adults in rural underserved communities with multiple disease preventive services simultaneously achieving various national and international health development goals.

## Introduction

Worldwide HIV/AIDS, diarrheal diseases, and malaria account for 165 million disability-adjusted life years lost per year, with HIV/AIDS the leading cause of death among adults in Africa.[1] Prevention and treatment of these diseases has posed enormous challenges in areas with limited health care infrastructure, shortage of health care staff, high disease burden, and widespread poverty. Western Kenya is a region with a high incidence of malaria,[2] diarrheal diseases,[3], [4] and HIV.[5] An average resident receives 150–300 malaria-infective mosquito bites per year,[2] with 5–15% HIV prevalence among adults.[5] The Government of Kenya has set a 2010 target of 80% of the population knowing their HIV status; yet currently only 36% of adults have ever had an HIV test, and less than 20% of adults living with HIV know that they are infected.[5] This is despite the HIV prevention benefit associated with knowing one's HIV status [6] including prevention of death and morbidity, such as HIV associated TB infection when antiretroviral treatment is commenced.[7]

Factors impeding expansion of HIV counseling and testing (HCT) include shortage of trained counselors, limited access to and high cost of transportation,[8] restricted test kit availability, and stigma. New programmatic approaches are needed if countries experiencing generalized HIV epidemics are to increase knowledge of status among the millions of infected adults and children currently at 20% among those living with HIV according to WHO.[9] Door-to-door HCT has had uptake of over 90% and offers the opportunity for couples to learn their status together.[5], [10] However, it is logistically challenging, time-intensive, and often the men and children were not at home, mostly out working or at school respectively.

Impediments to HCT uptake might be overcome by attracting people to sites through campaigns that provide other preventive health commodities in addition to HCT. Provision of a multi-disease prevention package (MPP) that contains preventive health commodities such as long lasting impregnated bednets (LLIN), a water purification system, preventive health education, condoms, and cotrimoxazole prophylaxis for adults living with HIV has been associated with reductions in morbidity and mortality.[11], [12] These interventions are recommended by WHO, and have been implemented in some programs in Africa. While this type of integrated response appears beneficial and cost-effective, it has only been offered to persons living with HIV, and has not been provided on a mass scale to people without HIV. Even though all components (except cotrimoxazole prophylaxis) would potentially benefit all recipients, including children for whom malaria and diarrheal diseases are among the leading five causes of morbidity and mortality in Kenya.[13] Large-scale integrated childhood immunization, mosquito net and de-worming programs have been successfully implemented,[14] but have not included HCT.

A multi-disease prevention campaign was planned and executed by the local community leadership, the Ministry of health Kenya (MoH), CHF International, CDC Kenya and Vestergaad Frandsen (VF) in partnership. The overall goal was to achieve the national target of 80% of adults aged 15–49 years knowing their HIV status and risk factors, providing and promoting the use of a MPP among all participants, and effectively linking persons living with HIV to care and treatment services.

## Methods

### Campaign design

The campaign was designed in collaboration with the local community leadership, the MoH Kenya, CHF International, CDC Kenya and VF. It was conducted in Lurambi division, Kakamega district, Kenya, covering 157 villages, an area of 194 km^2^ and estimated population of 115,802 according to the Kenya National Bureau of Statistics 2008 projections. The campaign goal was to test at least 40,900 of 51,178 individuals aged 15–49 years in 7-days. However, all persons above 15 residing within the district were attended to. A district coordinating committee mapped 30 sites within the division through which HCT, a MPP and health education were provided. Sites were selected according to their suitability and geographic location. A micro-planning exercise determined the numbers of clients that would be expected to attend each site on each day of the campaign to determine the personnel and logistics requirements for each site. The campaign was anticipated to last seven days. In addition, one mobile HCT truck was used to support sites during periods with high demand.

A pre-campaign survey was conducted two months prior to the campaign and used semi-structured questionnaires and focus groups to identify appropriate media messages and channels required to encourage residents aged 15–49 years to attend the campaign. An ongoing program of health education and community mobilization was conducted in the month preceding the campaign and during the campaign itself.

During the campaign, each participant was registered upon entering a site with demographic data collected using standard national HCT forms. They were given a unique client number each delinked from identifiers thus ensuring confidentiality. While waiting, groups of 20–40 people at a time received basic health education and instructions on how to use the MPP commodities. A nationally certified HCT counselor provided individual pre-test counseling and obtained verbal informed consent before conducting an HIV test. Each participant received a MPP following the HIV test, or following pre-test counseling if they declined an HIV test. Persons living with HIV received a three month supply of cotrimoxazole prophylaxis and were referred to appropriate care and treatment services.

### Procedures

HCT was provided according to national MoH guidelines. Finger prick blood samples were screened for HIV infection using two enzyme-linked immunoassays in parallel (Determine HIV-1/2, Inverness Medical, Princeton, NJ, USA; and Bioline HIV-1/2 3·0, Taylor Hor, Republic of Korea). Samples concordant positive or negative on both assays were not tested further. Those with discordant results were retested using a third tie-breaker (UniGold Recombigen, Trinity Biotech USA). A laboratory technician at each site provided quality assurance for testing. For quality control, 10% of dried blood spots collected on filter paper from participants were screened by EIA and reactive specimens confirmed by Vironostika HIV Uni-Form II Ag/Ab (BioMérieux, The Netherlands). CD4-count, white cell count and lymphocyte counts were determined for persons testing HIV positive at 2 randomly selected sites by portable Aurica flow cytometers using standard protocols (PointCare®, Massachusetts, USA).

### Data management and statistical analysis

Basic demographic and behavioral data were recorded on national HCT forms, entered and cleaned for errors and inconsistencies, and analyzed using SAS v9.13. (SAS Corp; Cary NC) and STATA v.10 (Stata Corp; College Station, TX). A multivariable logistic regression model was developed to assess association of HIV status with other factors. All variables significantly associated with HIV-infection in stratified univariate analyses were included in multivariable analyses to identify potential risk factors for HIV-infection in sexually active individuals. Multivariate odds ratios (ORs) and 95% confidence intervals (CIs) are presented unless otherwise stated. For all analyses two-sided tests were used; 95% CIs not including 1 were considered significant. The ORs by age were significantly different by gender; therefore those ORs are presented separately. Separate logistic regression models for men and women who were sexually active within the last 12 months are also presented. The stepwise Akaike Information Criterion was used for best model fit.

### Ethics statement

This was a public health program to expand access to HCT integrated with malaria and diarrhea prevention in line with set MoH National targets of achieving 80% of the sexually active population to know their status and other international millennium development goals 4, 5 and 6 thus did not require IRB and ethical approvals. A letter endorsing and authorizing this public health intervention in Western Kenya was obtained from the office of the Permanent secretary in the Ministry of Public Health and Sanitation Kenya.

To participate in the campaign and have an HIV test, all participants were registered in VCT national registers and verbal informed consent obtained in accordance with National Guidelines for HIV Testing and Counselling in Kenya (National AIDS and STD Control Programme (NASCOP) Ministry of Public Health and Sanitation, Kenya 2008.[15]

## Results

### Uptake of HIV Counseling and Testing

**Figure 1:**
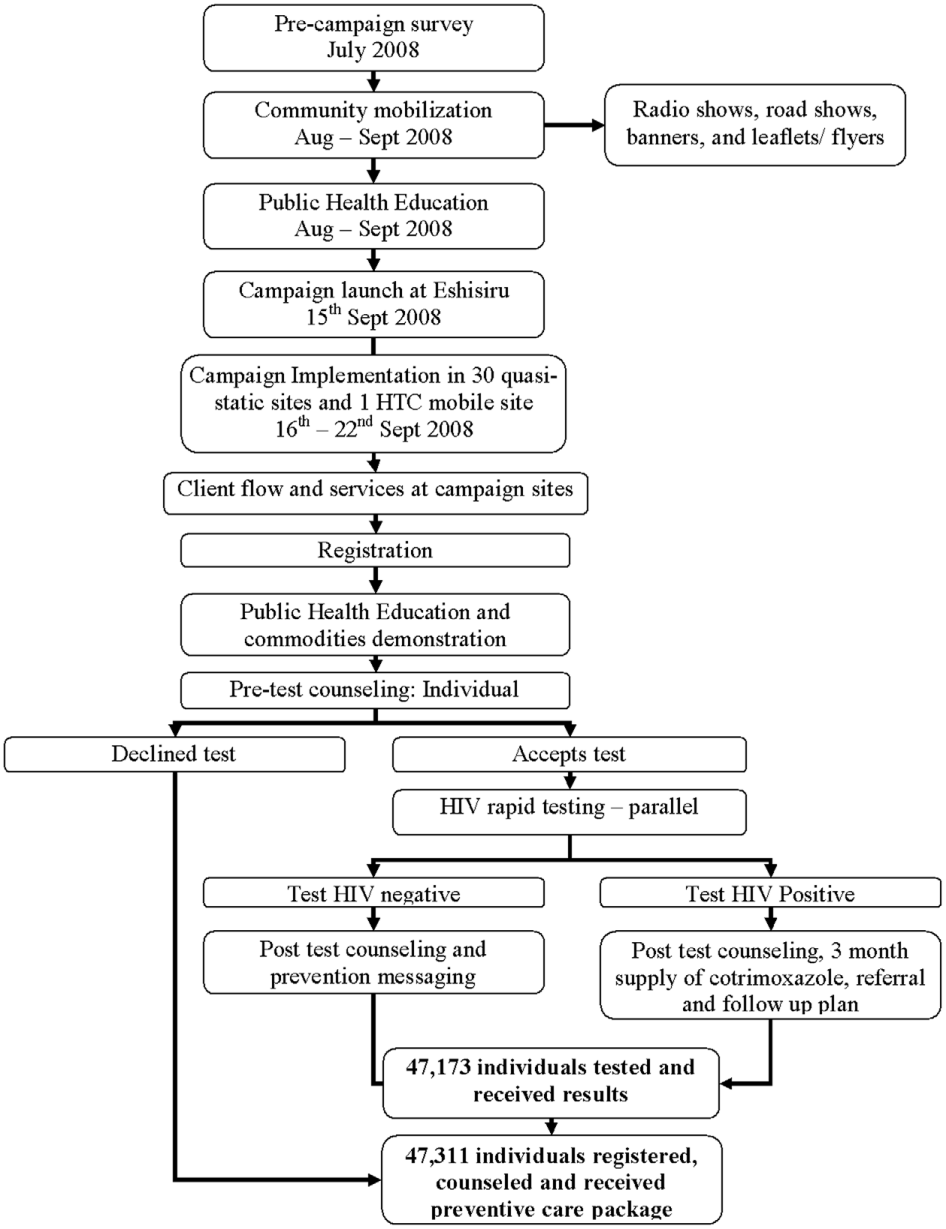
Campaign flow chart.

*Figure 1* shows the flow through each campaign site. The multi-disease preventive package (MPP) was provided to 45,418 (96%) of all registered participants, <1% were counseled but opted not to test, 1% required information only and 2% had missing data on the MPP uptake status. The average length of time spent by participants at the campaign site including registration, health education, counseling and testing, and receipt of the MPP was one hour (IQR: 0.5–6 hrs).

A total of 566 personnel implemented the campaign, including 386 certified HCT counselors, 35 laboratory technicians, 31 persons living with HIV, counselor supervisors, commodity distributors and zonal store keepers assigned to each of the 31 HCT sites. On average each counselor attended to a total of 122 individuals over a period of 7 days; counseling 15 to 17 persons per day.

### Demographic Characteristics

A total of 47, 311 individuals registered at the campaign, of these 47,173 (99·7%) were tested for HIV and received their results (*Table 1*). The median age of those tested was 30 years (IQR: 20, 43) for men and 30 years (IQR: 22, 40) for women; 5,683 (12%) were above 49 years of age.

**Table 1:** Demographics of participants tested for HIV in Lurambi Western Kenya, 2008 (N = 47173).

	Total at Risk	HIV infected	Bivariate Odds Ratio(95% CI)
**Gender**			
Women	28906	1448(5.0%)	1.0 [Ref]
Male	18101	508(2.8%)	0.55 (0.49–0.61)
Missing*	*166*	*8 (4.8%)*	
**Age group**			
<15 years	170	4(2.4%)	3.55 (1.27–9.88)
15–19	8307	65(0.8%)	1.0 [Ref]
20–24	8347	270(3.2%)	4.32 (3.27–5.71)
25–29	6225	322(5.2%)	6.87 (5.21–9.04)
30–34	5395	360(6.7%)	8.99 (6.84–11.81)
35–39	4699	316(6.7%)	9.41 (7.15–12.39)
40–44	3766	246(6.5%)	8.89 (6.70–11.80)
45–49	4384	204(4.7%)	6.06 (4.53–8.09)
50–55	2524	108(4.3%)	5.58 (4.05–7.67)
Above 55	3159	63(2.0%)	2.54 (1.77–3.64)
Missing*	*197*	*6(3.1%)*	
**Marital Status**			
Never Married	9577	106(1.1%)	1.0 [Ref]
Married, (mono) or Steady part, living together	26520	1084(4.1%)	3.81 (3.12–4.66)
Married, (poly)	4939	275(5.6%)	5.27 (4.20–6.61)
Sep/divorced	811	118(14.6%)	15.21 (11.58–20.00)
Steady partner not living together	2451	97(4.0%)	3.68 (2.79–4.87)
Widowed	2514	272(10.8%)	10.84 (8.62–13.63)
Missing*	*361*	*12(3.3%)*	*Fill in data*
**Condom use at last contact among sexually active**			
No	34454	1398 (4.1%)	1.0 [Ref]
Yes	3133	202 (6.5%)	1.66 (1.43–1.93)
Yes (broke)	85	5 (5.9%)	1.96 (0.90–4.23)
**Education**			
None	6196	243(3.9%)	1.0 [Ref]
Some primary	27462	1230(4.5%)	1.17 (1.01–1.35)
Some secondary	10292	365(3.6%)	0.93 (0.78–1.10)
Some post secondary	2857	105(3.7%)	0.97 (0.76–1.23)
Missing*	*366*	*21(5.7%)*	
**Occupation**			
None	13567	629(4.6%)	1.0 [Ref]
Professional	2157	82(3.8%)	0.82 (0.64–1.04)
Student	5465	20(0.4%)	0.07 (0.04–0.11)
Skilled worker	6967	342(4.9%)	1.06 (0.92–1.22)
Unskilled worker	18695	878(4.7%)	1.03 (0.92–1.15)
Missing*	*322*	*13(4.0%)*	
**Male circumcision**			
No	2010	82(4.1%)	1.0 [Ref]
Yes	15809	418(2.6%)	0.64 (0.50–0.81)
Don't Know	22	3 (13.6%)	3.71 (1.08–12.80)
*N/A (women)*	*29354*	*1464 (5.0%)*	
**Pregnant (women)**			
No	26057	1324(5.1%)	1.0 [Ref]
Yes	2550	110(4.3%)	0.84 (0.69–1.03)
Don't know	266	12(4.5%)	0.88 (0.49–1.58)
*N/A (men)*	*18,300*	*518(2.8%)*	
**Client seen as**			
Individual	41058	1750(4.3%)	1.0 [Ref]
Couple	3296	121(3.7%)	0.86 (0.71–1.03)
Group	2080	64(3.1%)	0.71 (0.55–0.92)
Polygamous group	126	1(0.8%)	0.18 (0.03–1.29)
Missing*	*613*	*28 (4.6%)*	
**Ever had sex?**			
No	3208	38 (1.2%)	1.0 [Ref]
Yes	42688	1871 (4.5%) (4.38%)	3.79 (2.73–5.26)
**Sex last 12 months**			
No	8141	298 (3.7%)	1.0 [Ref]
Yes	39032	1666 (4.3%)	0.85 (0.75–0.97)

A total of 1,964 (4%) of those tested for HIV were positive. HIV prevalence was 1,448 (5%) for women and 508 (3%) for men. Of those tested, 80% had never had an HIV test before and of these 4% tested HIV-positive. For 7,849 individuals who reported having had a previous HIV test, 4% reported a previous positive result, however 6% of these tested positive in this round. Of 7,374 individuals who reportedly had a previous HIV-negative result, 237 (3%) tested positive. A CD4+ cell count was determined for 113 (6%) of persons living with HIV at 2 HCT sites. The median CD4 cell count was 541 cell/µL (IQR; 356–754); 13 (11·5%) had a CD4 count of <250 cell/µL and were eligible for antiretroviral therapy (ART), according to national guidelines. 28 (25%) had a CD4 cell count <350 cells/µL.

### Diarrhea, malaria and STI prevention

There was a high uptake of commodities for malaria and diarrhea prevention during the campaign. All the MPPs distributed contained one LLIN and a supply of 60 male condoms. In addition, 51% MPP containing household filters were distributed to women, while men received 46% MPP containing individual filters. The water filter type was not indicated for 3% of participants.

### Multivariate Analysis

**Figure 2:**
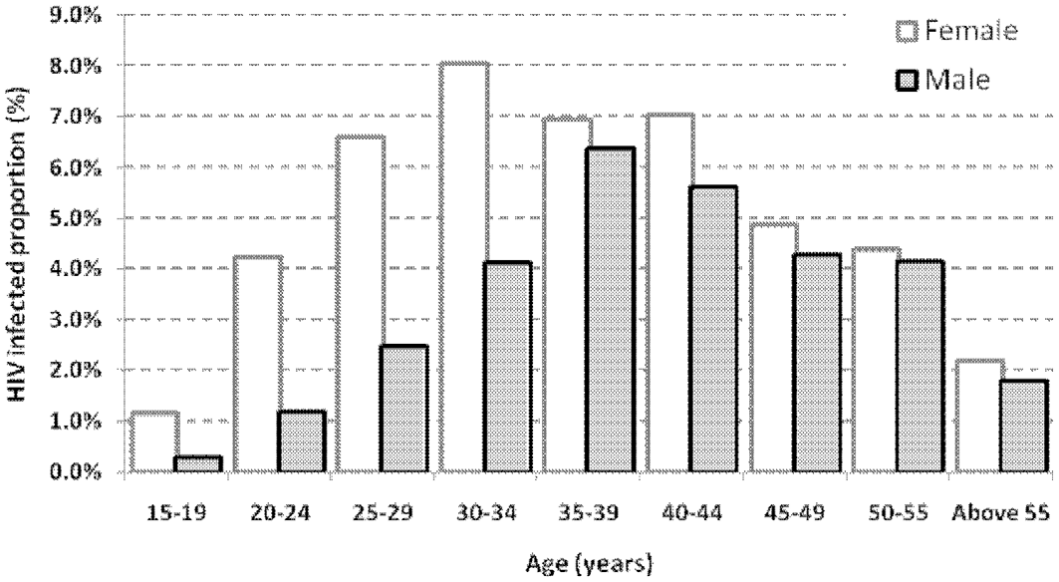
Distribution of HIV prevalence; Lurambi Division, Kenya 2008.

HIV prevalence by age group differed by gender (*Figure 2*), therefore all risk factor analyses were stratified by gender to control for effect modification. In bivariate analyses factors that showed an association with HIV-infection included age, current marital status, sexual encounter, male circumcision and occupation (*Table 2*). In a multi-variable analysis, current marital status (widowed vs never married, OR. 3·91; 95% CI. 2·87–5·34 and separated/divorced vs never married, OR. 4·43; 95% CI.3·13–6·27) and occupation (student vs no occupation, OR. 0·25; 95% CI.0·14–0·45) in women were significantly associated with HIV. In men, current marital status (widowed vs never married, OR. 7·01; 95% CI. 3·53–13·90 and separated/divorced vs never married, OR. 6·76; 95% CI.3·62–12·63) and occupation (student vs no occupation, OR. 0·18; 95% CI.0·05–0·61) were significantly associated with HIV. Potential factors not statistically associated with HIV-infection in the multivariable analysis included male circumcision and sex in the last 12 months.

**Table 2:** Risk factors for HIV infection among men and women in Lurambi Western Kenya 2008.

	Women			Men		
Variable	At risk	Infected (%)	Unadjusted OR (95% C.I)	At risk	Infected (%)	Unadjusted OR (95% C.I)
**Age**						
15–19	3773	40 (1.1%)	1.0 [Ref]	3517	9 (0.3%)	1.0 [Ref]
20–24	3894	163 (4.2%)	3.69 (2.72–5.01)	2217	25 (1.1%)	4.18 (2.11–831)
25–29	2873	187 (6.5%)	5.75 (4.24–7.80)	1685	43 (2.6%)	8.64 (4.50–16.60)
30–34	2564	190 (7.4%)	7.21 (5.32–9.76)	1511	59 (3.9%)	13.94 (7.38–26.35)
35–39	2324	137 (5.9%)	6.33 (4.64–8.64)	1317	74 (5.6%)	23.21 (12.43–43.29)
40–44	1962	123 (6.3%)	6.26 (4.55–8.62)	1117	59 (5.3%)	20.15 (10.67–38.03)
45–49	2293	95 (4.1%)	4.14 (2.98–5.76)	1358	53 (3.9%)	14.56 (7.67–27.65)
50–55	1279	47 (3.7%)	3.72 (2.54–5.45)	960	32 (3.3%)	14.16 (7.29–27.50)
Above 55	1469	32 (2.2%)	1.89 (1.22–2.93)	1390	18 (1.3%)	5.69 (2.79–11.59)
**Marital Status**						
Never Married	3667	66 (1.8%)	1.0 [Ref]	4618	20 (0.4%)	1.0 [Ref]
Married, (mono)	10632	440 (4.1%)	2.27 (1.79–2.87)	6978	222 (3.2%)	8.26 (5.39–12.64)
Married, (poly)	2917	154 (5.3%)	3.37 (2.60–4.37)	1071	34 (3.2%)	9.08 (5.53–14.91)
Sep/divorced	361	56 (15.5%)	11.18 (8.11–15.41)	250	23 (9.2%)	22.75 (12.93–40.03)
Steady part, living together	1806	84 (4.7%)	2.45 (1.82–3.29)	1052	34 (3.2%)	8.15 (4.91–13.52)
Steady partner not living together	1019	45 (4.4%)	2.61 (1.87–3.65)	800	19 (2.4%)	4.96 (2.71–9.07)
Widowed	1919	164 (8.6%)	6.30 (4.86–8.16)	215	18 (8.4%)	21.02 (11.46–38.56)
**Education**						
None	4035	162 (4.0%)	1.0 [Ref]	1375	29 (2.1%)	1.0 [Ref]
Some primary	13232	639 (4.8%)	1.22 (1.04–1.43)	8918	242 (2.7%)	1.35 (0.94–1.93)
Some secondary	4097	172 (4.2%)	1.04 (0.86–1.26)	3821	73 (1.9%)	1.08 (0.73–1.59)
Some post-secondary	954	37 (3.9%)	0.99 (0.74–1.33)	878	22 (2.5%)	1.32 (0.82–2.12)
**Occupation**						
None	8111	406 (5.0%)	1.0 [Ref]	2825	62(2.2%)	1.0 [Ref]
Professional	649	29 (4.5%)	0.93 (0.69–1.25)	710	17 (2.4%)	1.03 (0.66–1.61)
Student	2275	13 (0.6%)	0.10 (0.06–0.18)	2668	3 (0.1%)	0.04 (0.01–0.13)
Skilled worker	2517	130 (5.2%)	1.11 (0.93–1.31)	2733	94 (3.4%)	1.57 (1.18–2.08)
Unskilled worker	8785	431 (4.9%)	1.05 (0.93–1.18)	6061	196 (3.2%)	1.39 (1.07–1.79)
**Pregnant (women)**						
No	26057	1324(5.1%)	1.0 [Ref]			
Yes	2550	110(4.3%)	0.84 (0.69–1.03)			
Don't know	266	12(4.5%)	0.88 (0.49–1.58)			
**Circumcision (Men)**						
No				2010	82(4.1%)	1.0 [Ref]
Yes				15809	418(2.6%)	0.64 (0.50–0.81)
Don't know				22	3 (13.6%)	3.71 (1.08–12.80)
Ever had sex?						
No	1664	20 (1.2%)	1.0 [Ref]	1240	6 (0.5%)	1.0 [Ref]
Yes	20394	972 (4.8%)	3.14 (2.20–4.50)	13533	361 (2.7%)	6.68 (2.98–14.96)
Sex last 12 months						
No	2629	110 (4.2%)	1.0 [Ref]	1504	13 (0.9%)	1.0 [Ref]
Yes	24951	1271 (5.1%)	1.23 (1.01–1.50)	15828	481 (3.0%)	3.60 (2.07–6.25)

In a sub-group analysis, participants who knew their HIV status were approximately five times more likely to use condoms the last time they had sex than those living with HIV yet did not know their status. (Women: OR. 4·66; 95% CI.3·07–7·09), men: OR. 5·21; 95% CI.2·77–9·82).

Men living with HIV who knew their status were 13·0 (95% CI.6·11–27·55) times more likely to always use a condom with a steady partner than those infected yet did not know their status. No significant difference in condom use with non-steady partners was shown in men living with HIV who knew their status compared to those infected yet did not know their status 0·91 (95% CI.0-3·60)). Women living with HIV who knew their status were more likely to always use a condom with a non-steady partner (OR. 5·39; 95% CI.2·26–12·84,) than those infected yet did not know their status, no significant difference was shown in always using a condom with steady partners.

## Discussion

This integrated HCT and disease prevention campaign conducted in rural Kenya achieved 87% coverage among the estimated targeted population of the 15–49 years old in only 7 days. Integrated disease prevention campaigns are feasible and may leverage limited resources to alleviate high burden of diseases.[16] High HCT coverage was achieved with HCT counselor loads of 15–17 clients served per day with in national recommended optimum for mass HCT campaigns. High uptake of HCT during this campaign may have been due to; buy–in and involvement of the community leadership, delivery of services at convenient locations near participants' homes and concomitant distribution of free LLINs, water filters, and condoms. There was a higher uptake of the entire MPP than has been seen in social marketing campaigns for LLINs, safe water systems[17] and HCT, suggesting that combining multiple-disease interventions increases utilization of services.

High HCT acceptance is comparable to over 90% rates observed in home-based, door-to door testing interventions successfully implemented in Uganda[10], [18], [19] and Kenya[20], and were achieved in a considerably less time. Such initiatives represent alternative options for wide spread coverage and acceleration of access to HCT services a necessary entry point for prevention and care,[6] in regions with generalized HIV epidemics particularly Kenya where less than 20% of adults living with HIV know that they are infected.

The average national HIV prevalence in Kenya is 7%, yet 84% of those infected with HIV do not know their HIV status[5] emphasizing the need to expand HCT services. During the campaign 96% of persons living with HIV were offered a 3-month course of cotrimoxazole prophylaxis and were referred for ongoing care and treatment. Identified persons living with HIV at two of the sites where offered point-of-care CD4 cell count testing, they had relatively high CD4 counts with a median of 541cell/µL, providing an opportunity for early provision of care and treatment.

Age, current marital status and occupation were associated with HIV-infection. These results support findings from other studies.[21], [22] Women of all ages experienced a higher risk of HIV infection than men of the same age-group, and throughout their lives, men were more likely to have sexual partners outside of marriage. These differences highlight the potential benefit of focusing specific interventions on changing social norms, such as power differentials between men and women regarding sex and reproductive choice, social expectations regarding sex, and access to financial resources and education. The high rate of HIV infection among divorced/separated individuals may reflect some of these imbalances. The increased odds of HIV infection among widows may result from some of the same factors as well as a high risk for HIV transmission during end-stage HIV disease when an individual's viral load is high.[23]

Malaria is still a leading cause of childhood morbidity and mortality in Kenya.[3], [4] Several trials in Africa have shown that use of LLINs is effective in reducing morbidity and mortality.[24], [25] When an entire village uses them a mass community effect extends even to those who do not use them.[24], [26] Scaled-up control efforts which include free distribution of LLINs to rural Africa is recommended[24], [27], [28] but complete coverage is still uncommon. Mass LLINs campaigns target pregnant women and children less than 5 years of age. However, adults and children with HIV are at higher risk for clinical malaria and severe outcomes. Mass campaigns such as this enhance access for both adults and children, thus promote universal coverage.

Lack of access to safe drinking water and inadequate sanitation contributes to increased risk of diarrheal diseases and causes an estimated two million deaths per year in under 5 year olds.[29] The 2003 Kenya Demographic and Health survey indicated that 75% of Nyanza province population in Western Kenya lacked access to improved water supplies and reported the third highest diarrhea rates in the country.[30] Evidence indicates that simple, acceptable, low-cost interventions such as water filtration offers the most consistent and effective means of improving household water and risk of diarrhea diseases and associated deaths.[31]. The campaign rapidly increased access to a safe drinking water intervention.

Challenges faced in this campaign included maintaining efficiency and quality of service provision when dealing with a large population within a short time. Waiting times were sometimes longer than desirable during peak hours, further complicated by massive population turn up at the same time. In addition, scores of school children below the age of 15 turned up for the campaign but were turned away highlighting the need for youth in school targeted interventions. A further challenge was to ensure sufficient staffing, particularly trained and certified HCT counselors and laboratory technicians. Through buy-in and collaboration with the local community leadership, we were able to mobilize trained personnel from two provinces creating a shortage. However, we ensured that other HCT centers operated with skeleton staffing during the short period of 7 days. Staff shortage could be addressed by enhanced training efforts in future campaigns. Integrated multi-disease prevention benefit to the community is likely to decrease both burden and cost on existing health systems in the long run. We did not assess the success of clinic referral for participants living with HIV. This should be addressed in future campaigns, especially when dealing with larger populations such as would be found in entire districts and provinces.

This campaign presents an operational model for reaching the magnitude of HCT coverage required for many national goals and may help control the HIV epidemic.[32] It is time to consider changing our current approach to include ‘outside the facility’ services if we are to achieve health targets and a healthy population. Multi-disease integrated campaigns represent an important and effective modality to augment routine health services if we are to achieve rapid, high and equitable coverage of multiple health challenges. This model could be adapted to other underserved communities. This may well be one of the fastest approaches to achieve national health objectives spelt out in the Millennium Development Goals (MDGs).[33]
