# Correction: Rapid Implementation of an Integrated Large-Scale HIV Counseling and Testing, Malaria, and Diarrhea Prevention Campaign in Rural Kenya

**DOI:** 10.1371/annotation/cd255375-7cf9-4b56-a81f-ec57e360c472

**Published:** 2010-09-16

**Authors:** Eric Lugada, Debra Millar, John Haskew, Mark Grabowsky, Navneet Garg, Mikkel Vestergaard, James G. Kahn, Nicholas Muraguri, Jonathan Mermin

The 7th author's middle initial was inadvertently omitted. The correct name is: James G. Khan.

