# Correction: Rapid Implementation of an Integrated Large-Scale HIV Counseling and Testing, Malaria, and Diarrhea Prevention Campaign in Rural Kenya

**DOI:** 10.1371/annotation/b3e1590a-5814-4848-958e-2af40d318936

**Published:** 2011-06-02

**Authors:** Eric Lugada, Debra Millar, John Haskew, Mark Grabowsky, Navneet Garg, Mikkel Vestergaard, James Kahn, Nicholas Muraguri, Jonathan Mermin

The author's last name is misspelled in the correction (doi:10.1371/annotation/cd255375-7cf9-4b56-a81f-ec57e360c472). The correct spelling is: James G. Kahn. 

